# Supplementary material for: Inflammatory factors and risk of lung adenocarcinoma: a Mendelian randomization study mediated by blood metabolites
Source: Front Endocrinol (Lausanne). 2024 Aug 27;15:1446863. doi: 10.3389/fendo.2024.1446863 (PMC11384989; doi:10.3389/fendo.2024.1446863)
Supplement: Supplementary file 1 [file DataSheet1.zip › supplementary files/S2/GCST90200205/sensitivity-analysis.pdf]

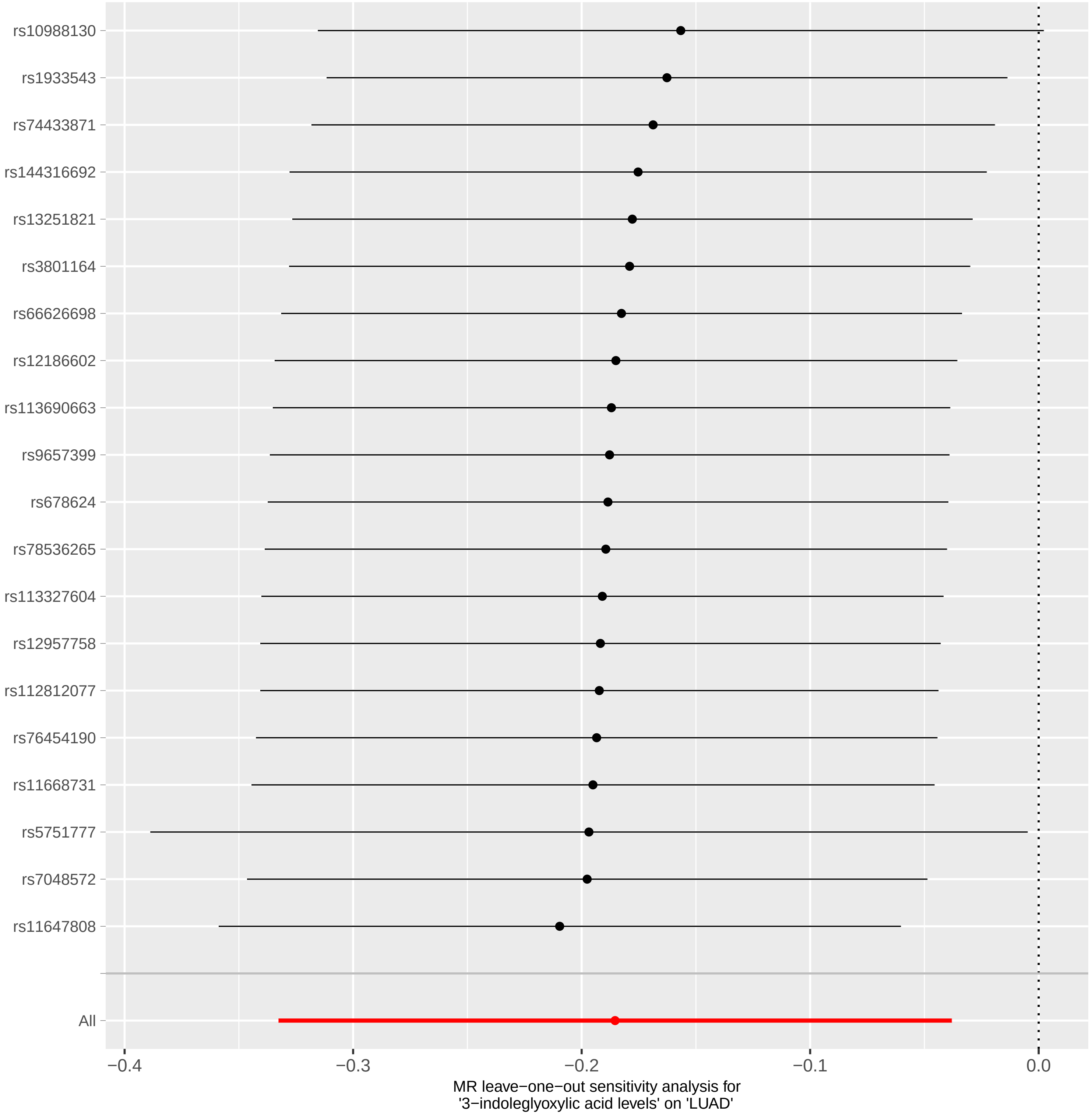

rs10988130
rs1933543
rs74433871
rs144316692
rs13251821
rs3801164
rs66626698
rs12186602
rs113690663
rs9657399
rs678624
rs78536265
rs113327604
rs12957758
rs112812077
rs76454190
rs11668731
rs5751777
rs7048572
rs11647808
All
−0.4
−0.3
−0.2
−0.1
0.0
MR leave−one−out sensitivity analysis for
'3−indoleglyoxylic acid levels' on 'LUAD'
